# Capacity Estimation Model for Signalized Intersections under the Impact of Access Point

**DOI:** 10.1371/journal.pone.0145989

**Published:** 2016-01-04

**Authors:** Jing Zhao, Peng Li, Xizhao Zhou

**Affiliations:** 1 Department of Transportation Engineering, University of Shanghai for Science and Technology, Shanghai, P.R. China; 2 Department of Civil Engineering and Mechanics, University of Wisconsin at Milwaukee, Milwaukee, Wisconsin, United States of America; 3 School of Economics and Management, Shanghai Maritime University, Shanghai, P.R. China; Beihang University, CHINA

## Abstract

Highway Capacity Manual 2010 provides various factors to adjust the base saturation flow rate for the capacity analysis of signalized intersections. No factors, however, is considered for the potential change of signalized intersections capacity caused by the access point closeing to the signalized intersection. This paper presented a theoretical model to estimate the lane group capacity at signalized intersections with the consideration of the effects of access points. Two scenarios of access point locations, upstream or downstream of the signalized intersection, and impacts of six types of access traffic flow are taken into account. The proposed capacity model was validated based on VISSIM simulation. Results of extensive numerical analysis reveal the substantial impact of access point on the capacity, which has an inverse correlation with both the number of major street lanes and the distance between the intersection and access point. Moreover, among the six types of access traffic flows, the access traffic flow 1 (right-turning traffic from major street), flow 4 (left-turning traffic from access point), and flow 5 (left-turning traffic from major street) cause a more significant effect on lane group capacity than others. Some guidance on the mitigation of the negative effect is provided for practitioners.

## Introduction

The access points affect traffic operations and safety by introducing conflicts and friction into the traffic stream [1]. It imposes a potential negative effect on signalized intersection capacity near signalized intersections.

Many previous works have been done to establish the adjustment factors of signalized intersection capacity, which is an important input for intersection optimization and signal timing [2–5]. In HCM2010 [6], the adjustment factors include lane width, heavy vehicles, grade, parking, bus blockage, area type, lane utilization, right turns, left turns, and pedestrians and bicycles. Other adjustment factors are also discussed, such as short-lane [7–9], weather condition [10], driving behavior [11–15], light condition [16], upstream weaving segment [17], adaptive cruise control system [18–21], advanced traveler information system [22–24], and advanced traffic management system [25, 26]. The change of the capacity is the result of car-following behavior. Extensive car-following models have been established including the consideration of factors to analyze the performance of the traffic system under different operation conditions. Those factors are geometric conditions [27–29], wrong way travel [30], shock wave [31], traffic flow stability [32], lateral separation [33], inter-vehicle communication [34, 35], electric vehicles [36], driver anticipation [37–40], optimal current difference, and anticipation optimal velocity. They are used to analysis the performance of the traffic system under different operation conditions [41–45].

Access management becomes one of the emerging themes in traffic engineering in recent years [46, 47]. Access management programs can smooth vehicle flow, reduce delay, and lead to fewer crashes. Technical constraints as well as political and institutional issues (e.g. the limits of land use, the width of the median and the density of road network), however, have limited widespread implementation, particularly in the developing countries. If it is not well addressed, the access point will be a potential effect factor to the capacity of signalized intersection. The previous research on the operational effects of access points mainly focused on the delays to drivers traveling along an arterial street segment and the capacity of access point itself based on the procedures for analyzing un-signalized intersections [48–50].

Although much is known about the operation of signalized intersections and the access point, little or no research has been conducted on the effect of access point on signalized intersection capacity to date. Even it was not considered as one of the adjustment factors in any of the capacity manuals. But if there is an access point near the signalized intersection, the demand starvation or queue spillback problem would be periodic and predictable.

In comparison with previous studies and applications, this paper:

1. Present a theoretical model for estimating the lane group capacity at signalized intersections with the consideration of the effects of access points.
2. Validate the proposed capacity model based on VISSIM simulation.
3. Provide practical guidance on the mitigation of the access point effect based on extensive numerical analyses.

The rest of the paper is organized as follows. Section 2 describes the framework of the computational procedure and the notation adopted in this paper. Section 3 discusses the traffic flow composition of an access point and the model of determining maximum throughput of the access point. Section 4 provides the formulations of the lane group capacity with the effect of access point. The proposed model is validated in Section 5. The effect of six types of access flow on the capacity of signalized intersection is analyzed based on numerical test in Section 6. Conclusions and recommendations are given at the end.

## Model Configuration

**Fig 1:**
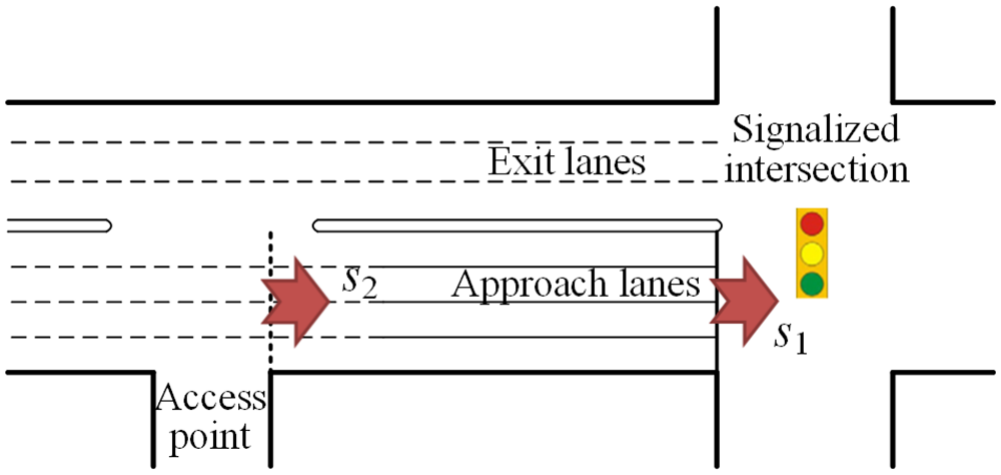
Scenario 1: Access point at upstream of intersection.

**Fig 2:**
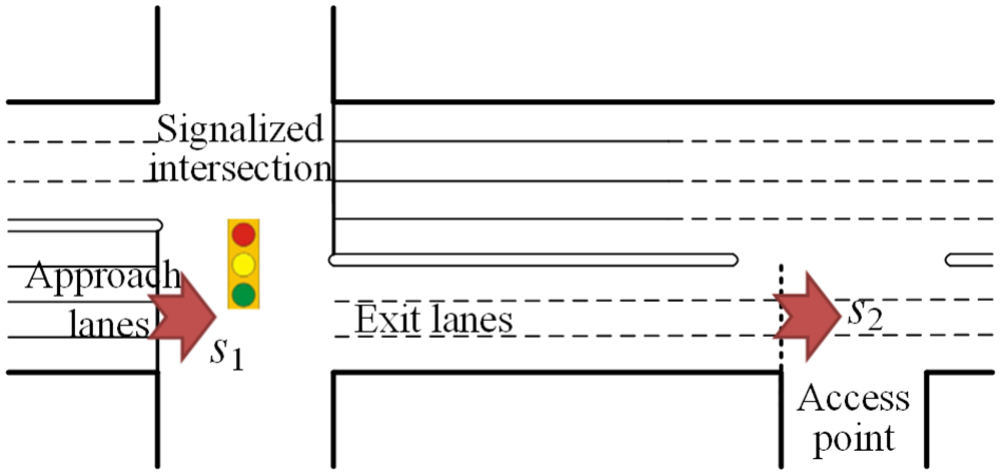
Scenario 2: Access point at downstream of intersection.

**Fig 3:**
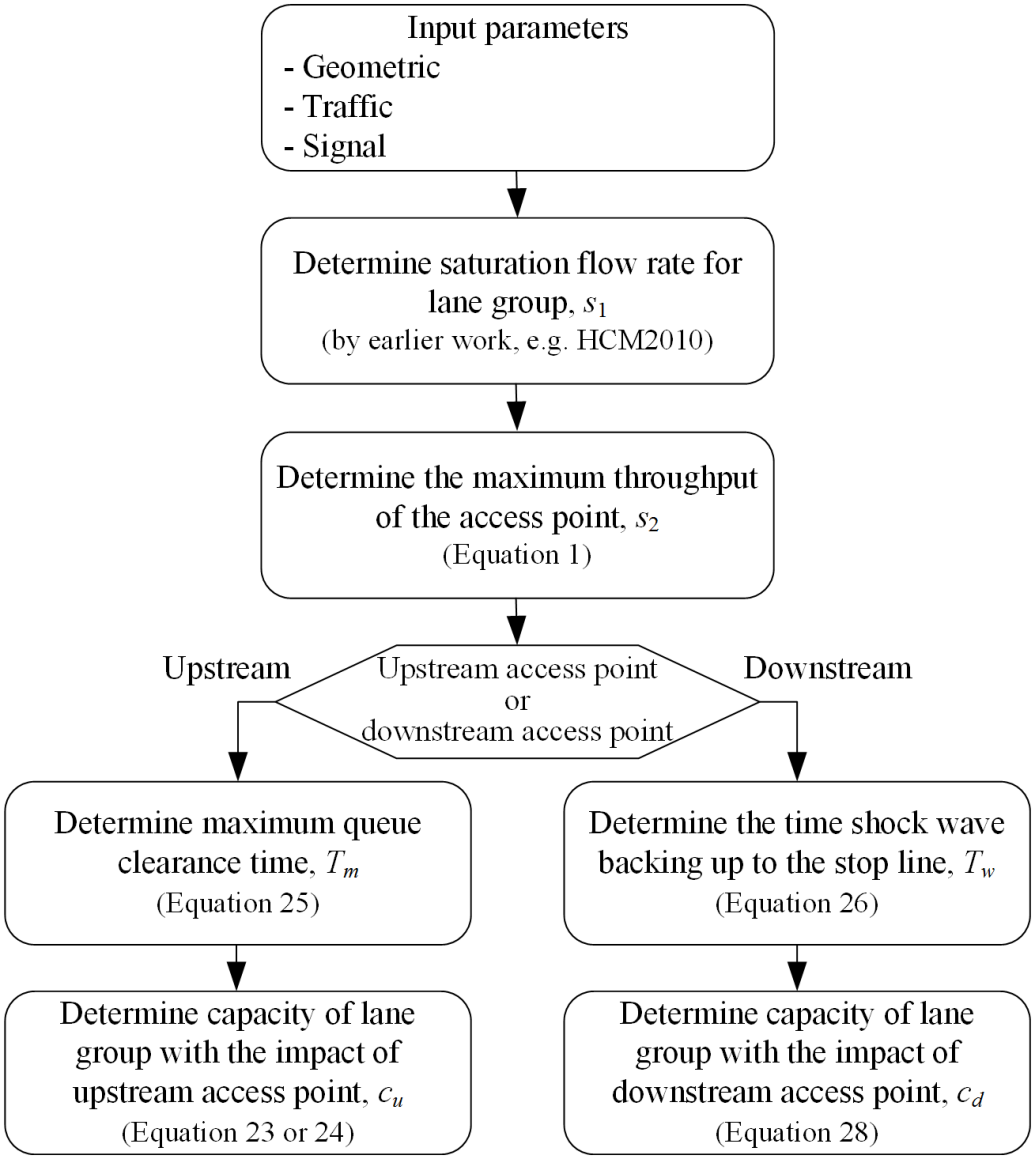
Outline of computational procedure.

Two scenarios of access point location are discussed in this paper as shown in Figs 1 and 2, respectively. The scenarios 1 is that the access point locates at upstream of the intersection. And the scenarios 2 is that the access point locates at downstream of the intersection. Wherever its location, the access point is a potential blockage. The paper discusses the traffic flow composition of an access point and the maximum through traffic flow rate at the access point (*s*_2_), firstly. Then the calculation model of the lane group capacity with the impacts of upstream and downstream access point is established, respectively. The outline of computational procedure is shown in Fig 3.

To facilitate model presentation, notations used hereafter are summarized in Table 1.

**Table 1:** Notation of key model parameters and variables.

*c*_*u*_	capacity of lane group with the impact of upstream access point (veh/h)
*c*_*d*_	capacity of lane group with the impact of downstream access point (veh/h)
*C*	cycle length (s)
*f*_*i*_	adjustment factor for the access traffic flow *i*
*g*_*e*_	effective green time for the lane group (s)
*G*_0*i*_	effective movement time of major street for access traffic flow *i* (s)
*G*_0*ei*_	green extension time of major street for access traffic flow *i* (s)
*G*_0*si*_	service time of major street under saturation flow rate for access traffic flow *i* (s)
*G*_*Ai*_	effective movement time for access traffic flow *i* (s)
*h*_*d*_	average space headway of stopped vehicle (m/veh)
*k*_*j*_	jam density (veh/m/ln)
*k*_*s*_	density of the saturation traffic flow (veh/m/ln)
*L*	distance between access point and stop line (m)
*N*	number of lanes
*N*_0_	average number of waiting vehicles (veh)
*N*_*B*_	number of blocked lanes
*p*(n)	probability of the queue vehicle number = n
*q*_02_	arrival rate of the outside lane of major street (veh/s)
*q*_04_	arrival rate of the inside lane of the opposing major street (veh/s)
*q*_06_	arrival rate of the inside lane of major street (veh/s)
*q*_*Ai*_	arrival rate of the access traffic *i* (veh/s)
*q*_*A*,*max*_	maximal traffic flow (capacity) of the access traffic (veh/s)
*q*_*M*_	arrival rate of all lanes of major street (veh/s)
*q*_*OM*_	arrival rate of the opposing major street (veh/s)
*r*_*e*_	effective red time for the lane group (s)
*s*_0_	base saturation flow rate per lane (veh/h/ln)
*s*_1_	saturation flow rate for subject lane group without the consideration of access point (veh/h)
*s*_2_	maximum throughput of the access point (veh/h)
*s*_*Ai*_	saturation flow rate for access traffic *i* (veh/s)
*t*_*c*_	critical time headways (s)
*t*_*f*_	move-up time (s)
*T*_*m*_	maximum queue clearance time (s)
*T*_*w*_	the length of time shock wave backing up to the stop line (s)
*u*_*w*_	speed of the shock wave (m/s)
*W*_*m*_	width of the median (m)
*Δ*	minimum arrival headway (s)
*φ*	proportion of free (un-bunched) vehicles

## Model Formulation

### Maximum throughput of the access point

**Fig 4:**
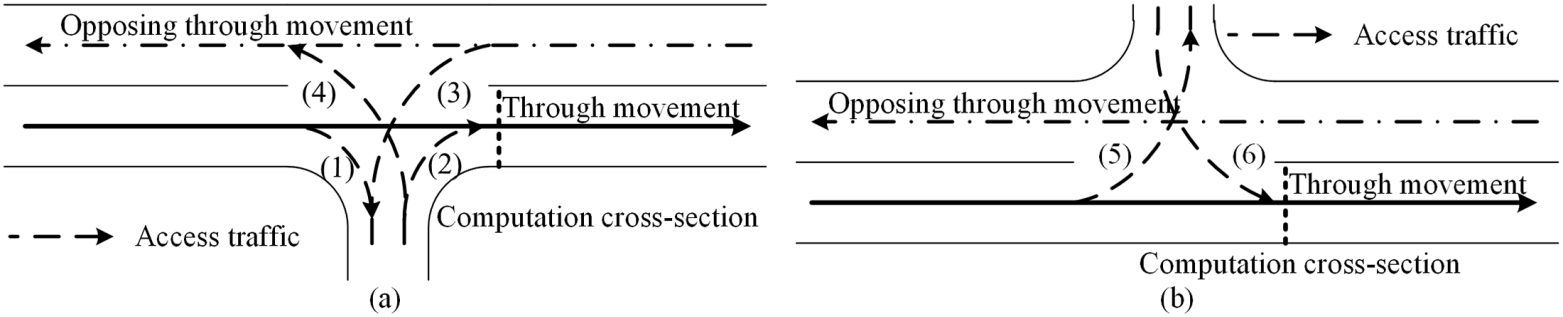
Composition of access point traffic flows. (a) Access point at right side of the road. (b) Access point at left side of the road.

**Fig 5:**
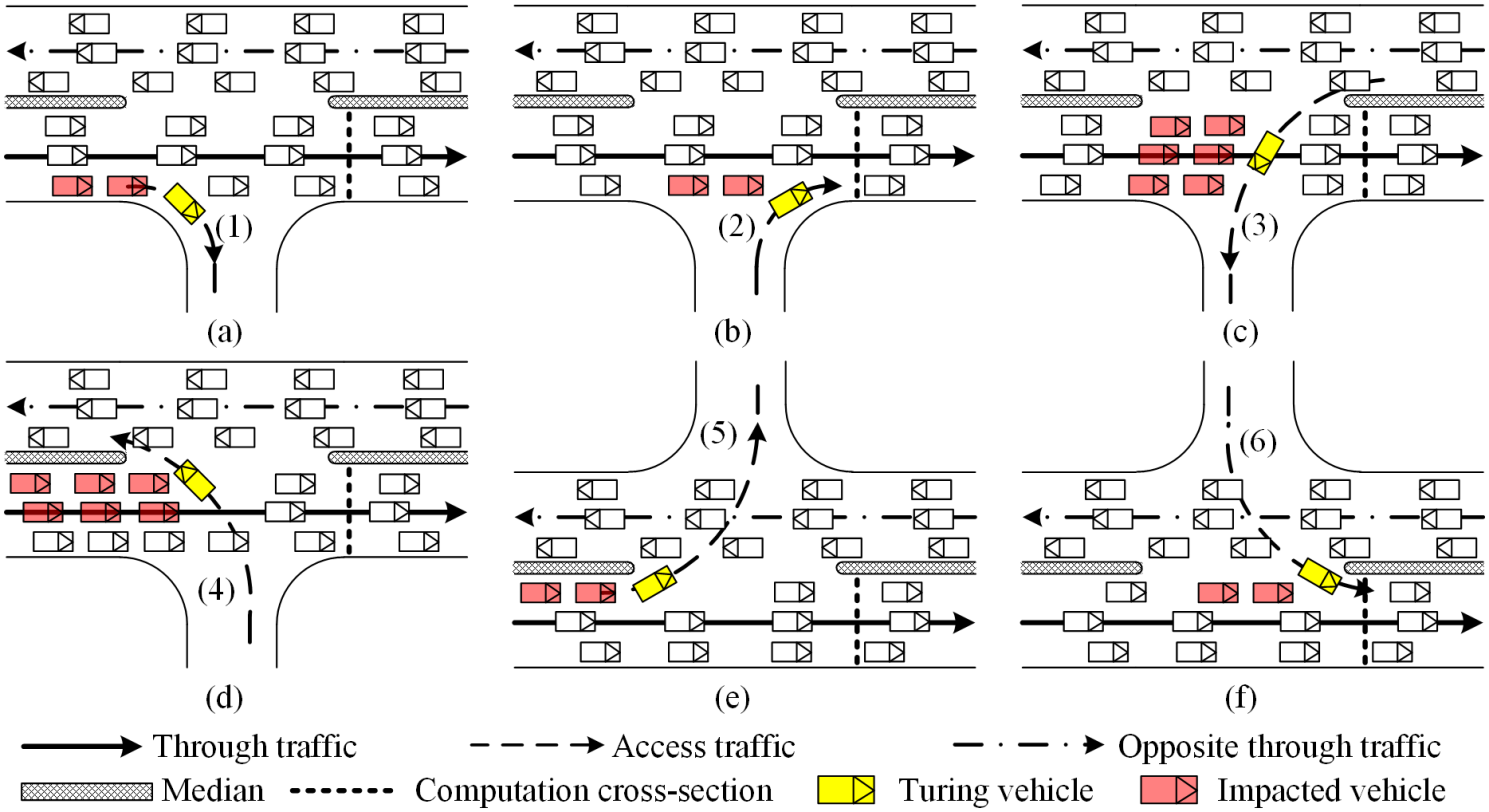
Impacts of access point traffic flows on the through traffic. (a) Access traffic flow (1). (b) Access traffic flow (2). (c) Access traffic flow (3). (d) Access traffic flow (4). (e) Access traffic flow (5). (f) Access traffic flow (6).

As illustrated in Fig 4, there are six types of access traffics. Their impacts on the maximum throughput are different, as shown in Fig 5. Eq 1 is used to compute the through traffic flow rate at the access point. The adjustment factors are described in the following subparts.

$$s_{2}=s_{0}Nf_{1}f_{2}f_{3}f_{4}f_{5}f_{6}$$ (1)

#### Adjustment for access traffic flow (1)

As illustrated in Fig 5(a), the access traffic flow (1) (right turn vehicles) often slow the following through vehicles when they enter into the access connection from the major street. So it will make the through traffic flow rate decrease, which can be illustrated as Eq 2. The proportion of right-turns and the decrease of saturation flow rate cause by right-turns are considered. The factor, 0.15, is based on the right turn adjustment formulation in HCM2010 [6].

$$\;f_{1}=1.0-0.15\frac{q_{A1}}{q_{M}}-\frac{q_{A1}}{q_{M}}$$ (2)

#### Adjustment for access traffic flow (2)

As Fig 5(b) illustrated, the access traffic flow (2) is allowed to enter the outside lane of the major street when an accepted gap occurs. It will cause the time loss of following through traffic when driving into the outside lane. The operation process of through traffic on a major street and the access traffic flow (2) is somewhat similar with the operation of traffic-actuated intersections [51]. Along the same lines as the capacity estimating model of traffic-actuated intersections, the adjustment factor for the access traffic flow (2) is calculated by Eq 3.

$$f_{2}=\frac{N-1+\frac{G_{02}+\frac{s_{A2}}{s_{02}}G_{A2}}{G_{02}+G_{A2}}}{N}$$ (3)

$$G_{02}=G_{0s2}+G_{0e2}$$ (4)

$$G_{0s2}=\frac{q_{02}G_{A2}}{s_{0}-q_{02}}$$ (5)

$$G_{A2}=\frac{q_{A2}G_{02}}{s_{A2}-q_{A2}}$$ (6)

$$G_{0e2}=\frac{e^{\frac{\varphi q_{02}}{1-\Delta q_{02}}\left(t_{c}-\Delta \right)}}{\varphi q_{02}}-\frac{1-\Delta q_{02}}{\varphi q_{02}}$$ (7)

#### Adjustment for access traffic flow (3)

The impact of access traffic flow (3) is that a part of time in which the conflict zone is occupied by it. So the proportion of time which is used by the through traffic should be determined. The operation principal of through traffic and the access traffic flow (3) at the conflict zone is similar to that of access traffic flow (2). The difference is, in this case, all lanes of the through traffic are impacted by access traffic flow (3), as illustrated in Fig 5(c).

$$f_{3}=\frac{G_{03}}{G_{03}+G_{A3}}$$ (8)

$$G_{03}=G_{0s3}+G_{0e3}$$ (9)

$$G_{0s3}=\frac{q_{M}G_{A3}}{s_{0}-q_{M}}$$ (10)

$$G_{A3}=\frac{q_{A3}G_{03}}{s_{A3}-q_{A3}}$$ (11)

$$G_{0e3}=\frac{e^{\frac{\varphi q_{M}}{1-\Delta q_{M}}\left(t_{c}-\Delta \right)}}{\varphi q_{M}}-\frac{1-\Delta q_{M}}{\varphi q_{M}}$$ (12)

#### Adjustment for access traffic flow (4)

As Fig 5(d) illustrated, the access traffic flow (4) has to cross the through traffic and merge into the inside lane of the opposing through traffic. If the median of the street is not wide enough, the through traffic of the major street is blocked by the access traffic flow (4) when it is waiting for the gap acceptance of the opposing through traffic. It can be illustrated by Eq 13, in which the average number of waiting vehicles is calculated based on the average queue length formulation at un-signalized intersections [52], as shown in Eq 14. The number of blocked lanes is related with the width of the median, as Eq 15 illustrated.

$$f_{4}=1-\frac{N_{0}N_{B}}{N}$$ (13)

$$N_{0}=\frac{{\left(\frac{q_{A4}}{q_{A,max}}\right)}^{\frac{1}{1+0.45\frac{t_{c}-t_{f}}{t_{f}}q_{04}}}}{1-{\left(\frac{q_{A4}}{q_{A4,max}}\right)}^{\frac{1}{1+0.45\frac{t_{c}-t_{f}}{t_{f}}q_{04}}\cdot \frac{1.51}{1+0.68\frac{t_{c}-t_{f}}{t_{f}}q_{04}}}}$$ (14)

$$N_{B}\;=\;\{\begin{array}{ccccc} 0, & & W_{m} & \geq & 5\\ 1,2 & \leq & W_{m} & < & 5\\ 2,0 & < & W_{m} & < & 2 \end{array}$$ (15)

#### Adjustment for access traffic flow (5)

As Fig 5(e) illustrated, the access traffic flow (5) cannot across opposing through traffic until accepted gap occurs. During the waiting time, it will impact the following through traffic flow. So the waiting time of left turn vehicles should be taken into consideration, which can be specified as Eq 16. The probability of no waiting vehicle can be calculated based on the queue length probability model at un-signalized intersections [52], as shown in Eq 17.

$$f_{5}=1-\frac{p\left(n\neq 0\right)}{N}$$ (16)

$$p\left(n\neq 0\right)={\left(\frac{q_{A5}}{q_{A5,max}}\right)}^{\frac{1}{1+0.45\frac{t_{c}-t_{f}}{t_{f}}q_{OM}}}$$ (17)

#### Adjustment for access traffic flow (6)

As Fig 5(f) illustrated, the access traffic flow (6) is allowed to enter the inside lane of the major street when an accepted gap occurs, so that it will cause the time loss of the following through traffic, which is similar to that of access traffic flow (2). Along the same lines as the adjustment factor estimating model of access traffic flow (2), the adjustment factor for the access traffic flow (6) is calculated by Eq 18.

$$f_{6}=\frac{N-1+\frac{G_{06}+\frac{s_{A6}}{s_{06}}G_{A6}}{G_{06}+G_{A6}}}{N}$$ (18)

$$G_{06}=G_{0s6}+G_{0e6}$$ (19)

$$G_{0s6}=\frac{q_{06}G_{A6}}{s_{0}-q_{06}}$$ (20)

$$G_{A6}=\frac{q_{A6}G_{06}}{s_{A6}-q_{A6}}$$ (21)

$$G_{0e6}=\frac{e^{\lambda \left(t_{c}-\Delta \right)}}{\varphi q_{06}}-\frac{1-\Delta q_{06}}{\varphi q_{06}}$$ (22)

### Capacity with the impact of upstream access point

**Fig 6:**
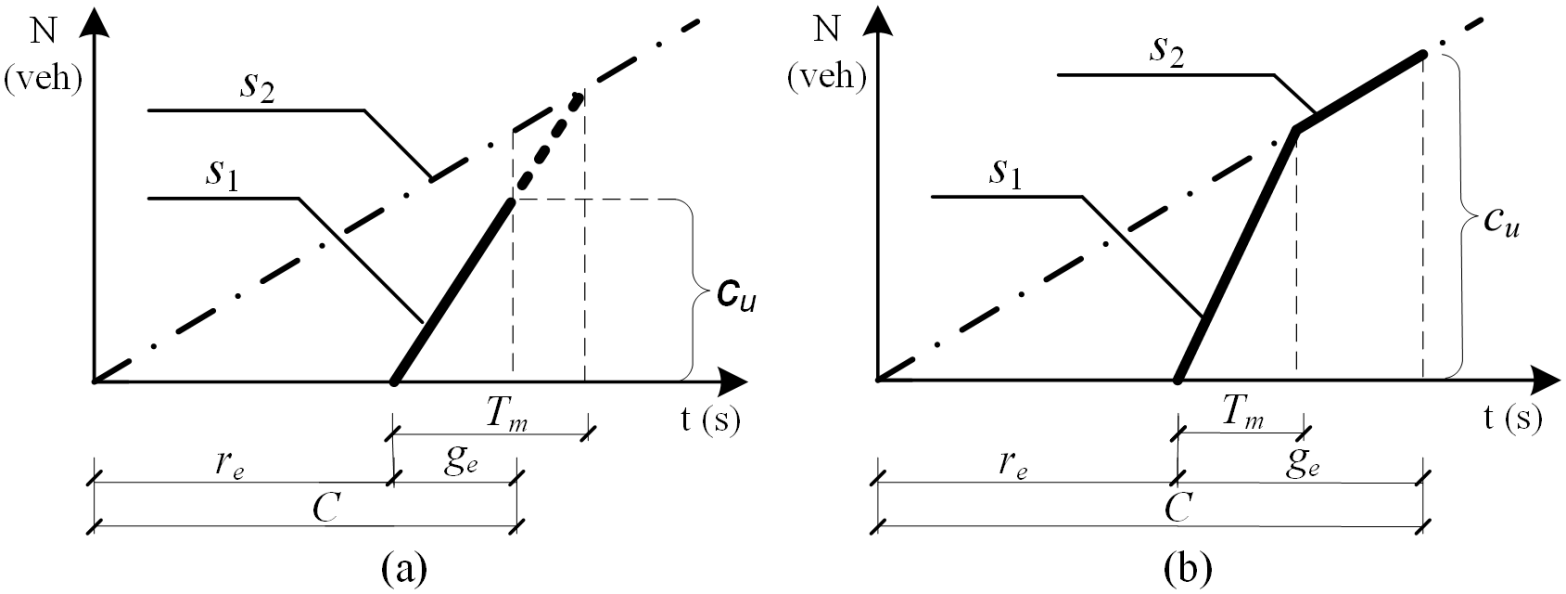
Queue accumulation polygons illustrating the effect of upstream access point. (a) Saturation 1: *g*_*e*_ ≦ *T*_*m*_. (b) Saturation 2: *g*_*e*_ > *T*_*m*_.

The upstream access point would cause a negative effect on approach capacity by the potential impact of upstream blockage. For example, when the input volume of the approach is limited by the upstream access point, the lane group capacity couldn’t be larger than the through traffic flow rate of the access point. The influence extent is related to the effective green time (*g*_*e*_) and the maximum queue clearance time (*T*_*m*_), which can be divided into two different situations, as shown in Fig 6.

As illustrated in Fig 6(a), when the effective green time is shorter than the maximum queue clearance time (*g*_*e*_ ≦ *T*_*m*_), the flow rate of the lane group is equal to the ideal saturation flow rate (*s*_1_), during all effective green time and the upstream access point has no effect on the lane group capacity. The capacity of a given lane group may be stated as shown in Eq 23.

$$c_{u}=s_{1}\frac{g_{e}}{C},\;\left(g_{e}\leq T_{m}\right)$$ (23)

As illustrated in Fig 6(b), when the effective green time is longer than the maximum queue clearance time (*g*_*e*_ > *T*_*m*_), the flow rate of the lane group is equal to the ideal saturation flow rate (*s*_1_) during the maximum queue clearance time, and it is equal to the ideal arrival rate of the approach (*s*_2_) during the rest of the effective green time. The capacity of a given lane group may be stated as shown in Eq 24.

$$c_{u}=s_{1}\frac{T_{m}}{C}+s_{2}\frac{g_{e}-T_{m}}{C},\;\left(g_{e}>T_{m}\right)$$ (24)

For a given signal timing, the maximum queue clearance time is computed using Eq 25. If the distance between an access point and stop line is long enough, the queue length of the arrival vehicles would not overflow to the access point, then the maximum queue clearance time is equal to the time for clearing the maximum arrival vehicles during the effective red time. Contrarily, if it does cause queue blockages to the access point, the maximum queue clearance time is equal to the time for clearing the stopped vehicles between the access point and stop line.

$$T_{m}=\{\begin{array}{c} \frac{s_{2}r_{e}}{s_{1}-s_{2}},\;\;L\geq \frac{s_{2}r_{e}h_{d}}{3600N}\\ \frac{3600LN}{s_{1}h_{d}},\;\;L<\frac{s_{2}r_{e}h_{d}}{3600N} \end{array}$$ (25)

### Capacity with the impact of downstream access point

**Fig 7:**
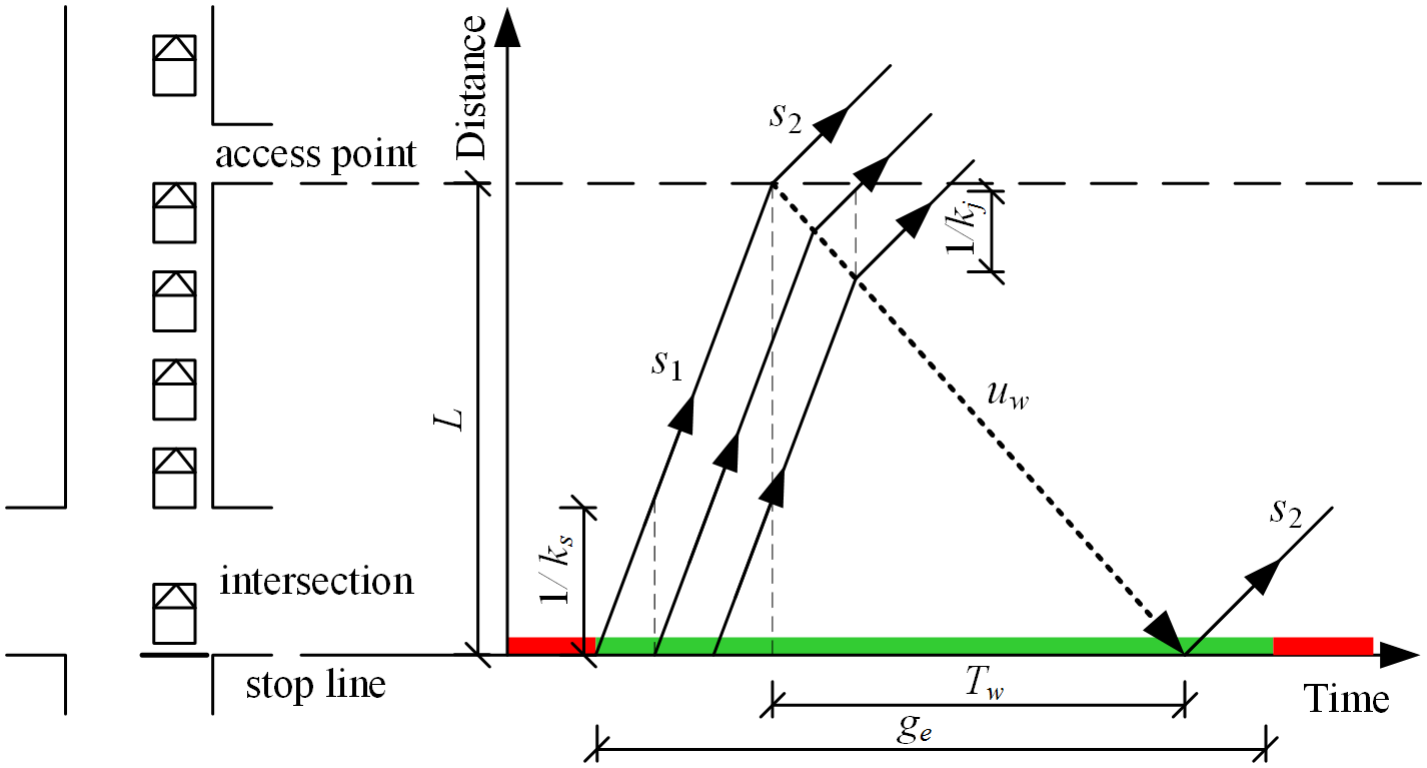
Shockwave dynamics illustrating the effect of downstream access point.

As illustrated in Fig 7, during the green time, traffic flow is going through the stop line with ideal saturation flow rate (*s*_1_). The departure rate of the receiving lanes is *s*_2_, which equal to the maximums through traffic flow rate at the downstream access point. If (*s*_1_ > *s*_2_), the access point can be considered as a traffic bottleneck at the downstream of the signalized intersection. The congestion will back up to the intersection, which greatly effects the operation of the approach [53]. The length of time shock wave backing up to the stop line can be determined based on shockwave dynamics theory [54], as Eqs 26 and 27 illustrated. Then, the capacity of a given lane group with the impact of downstream access point may be stated as shown in Eq 28.

$$T_{w}=\frac{L}{u_{w}}$$ (26)

$$u_{w}=\frac{s_{1}-s_{2}}{3600\left(k_{j}-k_{s}\right)}$$ (27)

$$c_{d}=\{\begin{array}{c} s_{1}\frac{g_{e}}{C},g_{e}\leq T_{w}\\ s_{1}\frac{T_{w}}{C}+s_{2}\frac{g_{e}-T_{w}}{C},\;g_{e}>T_{w} \end{array}$$ (28)

## Model Validation

In this section, the proposed capacity model is validated based on VISSIM simulation. Table 2 summarized the data inputs. Totally, 72 scenarios were tested. Model validation results are shown in Table 3. As Table 4 illustrated, the paired samples T-test shows that there was no significant difference between the capacity calculated by the proposed model and that obtained by the simulations (t = 1.100, P = 0.275 > 0.05). Consequently, the accuracy of the proposed capacity estimation models is acceptable.

**Table 2:** Model validation cases.

Input data	Number of cases	Value
Location of the access point	4	Case 1: upstream + right side of the road, Case 2: upstream + left side of the road, Case 3: downstream + right side of the road, Case 4: downstream + left side of the road
Number of lanes	3	1, 2, 3
Distance between access point and stop line (m)	6	50, 60, 70, 80, 90, 100
Cycle length (s)	1	120
Effective green time (s)	1	32
Effective red time (s)	1	88
Flow rate ratio between major street and access traffic	1	3:1

**Table 3:** Model validation results.

Location of the access point	*N*	*L*	Model calculation results (veh/h)	VISSIM simulation results (veh/h)	Location of the access point	*N*	*L*	Model calculation results (veh/h)	VISSIM simulation results (veh/h)
upstream + right side of the road	1	50	1166	1103	downstream + right side of the road	1	50	1351	1248
		60	1277	1237			60	1517	1406
		70	1406	1525			70	1684	1821
		80	1573	1713			80	1813	1678
		90	1665	1787			90	1832	1912
		100	1795	1823			100	1850	1864
	2	50	2886	2743		2	50	3367	3266
		60	2997	2832			60	3552	3468
		70	3145	3002			70	3663	3803
		80	3367	3636			80	3700	3725
		90	3478	3674			90	3700	3727
		100	3626	3686			100	3700	3727
	3	50	4773	4558		3	50	5384	5344
		60	4884	4655			60	5495	5453
		70	4995	4734			70	5550	5512
		80	5217	5082			80	5550	5509
		90	5328	5444			90	5550	5595
		100	5495	5584			100	5550	5593
upstream + left side of the road	1	50	1425	1354	downstream + left side of the road	1	50	1462	1356
		60	1499	1442			60	1573	1483
		70	1591	1645			70	1684	1695
		80	1684	1637			80	1813	1737
		90	1739	1805			90	1832	1840
		100	1813	1840			100	1850	1872
	2	50	3256	3117		2	50	3441	3334
		60	3330	3262			60	3589	3495
		70	3404	3547			70	3663	3770
		80	3515	3675			80	3700	3729
		90	3589	3727			90	3700	3724
		100	3663	3740			100	3700	3731
	3	50	5106	4939		3	50	5439	5382
		60	5162	4969			60	5495	5398
		70	5273	5038			70	5550	5512
		80	5384	5170			80	5550	5508
		90	5439	5590			90	5550	5588
		100	5495	5596			100	5550	5591

**Table 4:** Paired samples test.

	Paired Differences					t	df	Sig. (2-tailed)
	Mean	Std. Deviation	Std. Error Mean	95% Confidence interval of the difference				
				Lower	Upper			
Pair: model—simulation	14.875	114.725	13.520	-12.084	41.834	1.100	71	0.275

## Sensitivity Analysis

In this section, the effect of six types of access flow on the capacity of signalized intersection is evaluated based on numerical analysis. Cycle length was set to 120 s in all cases. Effective green time and red time for the lane group were set to 32 s and 88 s, respectively. Average space headway of stopped vehicle was set to 7 m. Arrival rate of each lane on major street was set to 300 veh/h/ln. Arrival rate of the access traffic was set to 100 veh/h. Jam density was set to 0.125 veh/m/ln. Density of the saturation traffic flow was set to 0.1 veh/m/ln. Base saturation flow rate per lane for major street was set to 1800 veh/h/ln. Lane group saturation flow rate for subject without the consideration of access point was set to 1650 veh/h/ln. Saturation flow rate for access traffic was set to 900 veh/h/ln. Critical time headways was set to 7.5 s. Move-up time was set to 4 s. Minimum arrival headway was set to 1.5 s. Proportion of free vehicles was set to 0.844. Width of the median was set to 0 m.

**Fig 8:**
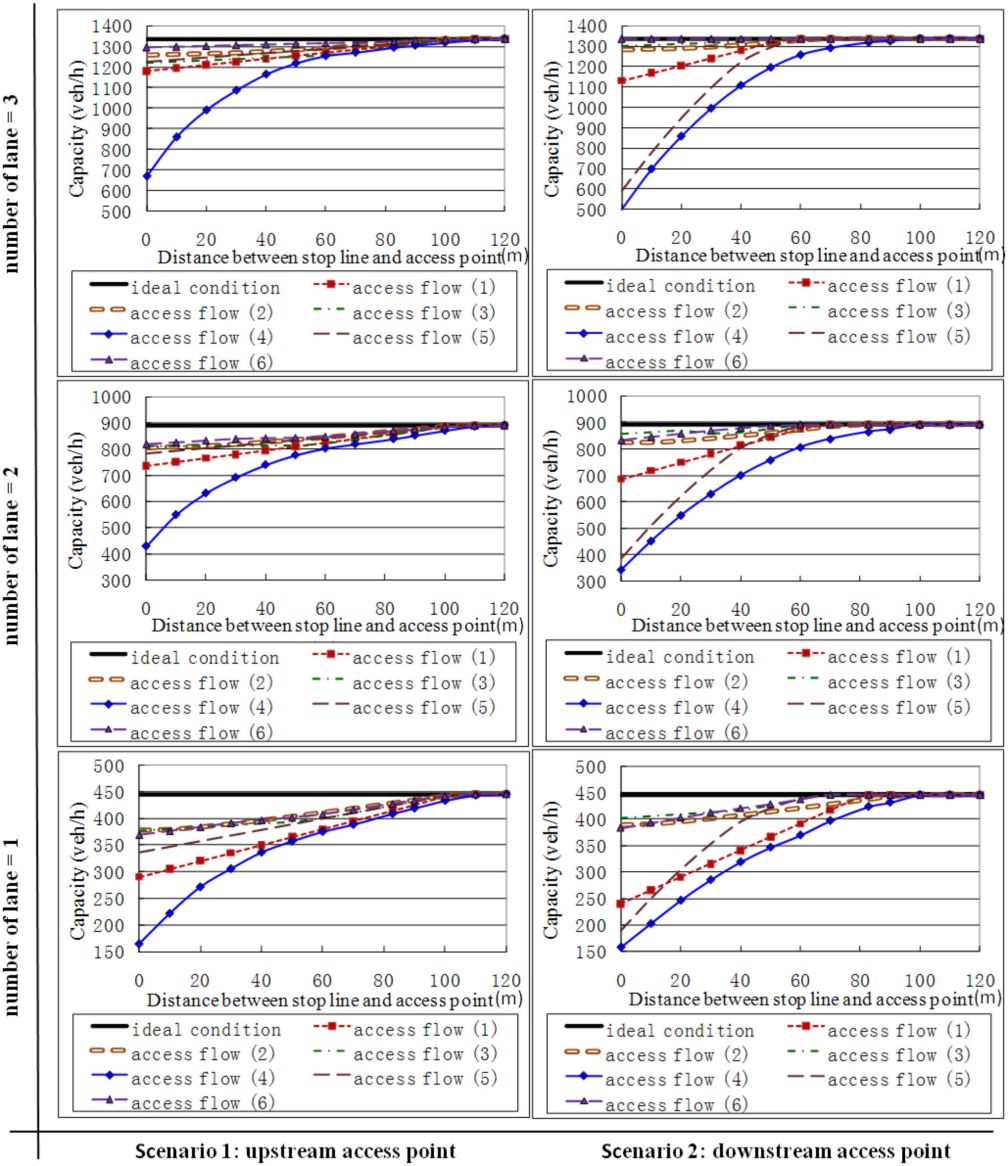
Comparison of capacities under the impact of different access traffic flows.

Fig 8 shows the analysis results, which includes six sub-pictures. In this Figure, the two columns respect the upstream access point situation (the left column) and downstream access point situation (the right column), respectively, and the number of major street lanes increases from bottom to top. For each sub-picture, the horizontal axe represents distance between stop line and access point, and the vertical coordinate is the lane group capacity of the intersection. The following observations could be made from Fig 8.

1. Overall, capacity increases with the distance between stop line and access point. This increase has an asymptotic shape towards the capacity of ideal condition.
2. If the distance between the intersection and access point is long enough, the effect of access traffic flow could be ignored. For example, when the cycle length equals to 120 s and the green time ratio equals to 0.27, the access point locating more than 100 m upstream of the intersection or 90 m downstream of the intersection has no effect to the intersection capacity.
3. The column sub-pictures show an inverse correlation between the number of major street lanes and the negative impact of access points on the capacity. The negative impact of access points fade away, but could not be eliminated, with the increase of the number of lanes on major street.
4. The access traffic flow 1 (right-turning traffic from major street), flow 4 (left-turning traffic from access point), and flow 5 (left-turning traffic from major street) cause a significant effect on lane group capacity. Therefore, auxiliary turning lanes should be used to reduce the severity and duration of conflict between turning vehicles and through traffic, if the land use is permitted.

## Conclusions

A theoretical model for estimating the lane group capacity with the consideration of the effects of access points is developed. From extensive numerical analysis, the following conclusions can be drawn:

1. The access point reduces the capacity of the lane group when it is close to the signalized intersection. The influence extent is mainly related to the signal timing, the number of lanes, the distance between the intersection and access point, and the types of access flows.
2. The access flows have no effect on lane group capacity if only the effective green time is shorter than the maximum queue clearance time in the case of upstream access point, or the effective green time is shorter than the time shock wave backing up to the stop line in the case of downstream access point.
3. Overall, the larger the number of major street lanes or the longer distance between the intersection and access point the less the negative impact of access point on the capacity generates. If the distance between the intersection and access point is long enough, the effect of access traffic flow could be ignored. For example, when the cycle length equals to 120 s and the green time ratio equals to 0.27, the access point locating more than 100 m upstream of the intersection or 90 m downstream of the intersection has no effect to the intersection capacity.
4. The access traffic flow 1 (right-turning traffic from major street), flow 4 (left-turning traffic from access point), and flow 5 (left-turning traffic from major street) have a significant effect on lane group capacity. Auxiliary turning lanes could be used to reduce those effects if the land use is permitted.

Some factors in the model, such as critical time headways, move-up time, minimum arrival headway, proportion of free vehicles, are closely related with the driver behavior [55–58] and local traffic condition [59, 60]. For application in the practice, they should be calibrated for local traffic condition.

## Supporting Information

S1 Table Lane group capacity under the impact of access traffic flows. (DOCX)
