# Supplementary material for: Capacity Estimation Model for Signalized Intersections under the Impact of Access Point
Source: PLoS One. 2016 Jan 4;11(1):e0145989. doi: 10.1371/journal.pone.0145989 (PMC4699772; doi:10.1371/journal.pone.0145989)
Supplement: S1 Table — (DOCX) [file pone.0145989.s001.docx]

**S1 Table. Lane group capacity under the impact of access traffic flows.**

|  | Distance between stop line and access point | | | | | | | | | | | |
| --- | --- | --- | --- | --- | --- | --- | --- | --- | --- | --- | --- | --- |
|  | 0 | 10 | 20 | 30 | 40 | 50 | 60 | 70 | 90 | 100 | 110 | 120 |
|  | Case 1: upstream access point, the number of lanes = 1 | | | | | | | | | | | |
| Ideal condition | 446 | 446 | 446 | 446 | 446 | 446 | 446 | 446 | 446 | 446 | 446 | 446 |
| Access flow (1) | 291 | 305 | 320 | 335 | 350 | 365 | 380 | 395 | 414 | 425 | 440 | 446 |
| Access flow (2) | 377 | 379 | 383 | 389 | 396 | 403 | 411 | 419 | 429 | 434 | 442 | 446 |
| Access flow (3) | 377 | 380 | 383 | 387 | 390 | 393 | 400 | 410 | 423 | 431 | 441 | 446 |
| Access flow (4) | 166 | 223 | 272 | 306 | 337 | 357 | 375 | 389 | 409 | 419 | 433 | 443 |
| Access flow (5) | 337 | 347 | 358 | 368 | 379 | 389 | 399 | 410 | 423 | 431 | 441 | 446 |
| Access flow (6) | 369 | 376 | 384 | 391 | 396 | 401 | 408 | 416 | 427 | 433 | 442 | 446 |
|  | Case 2: downstream access point, the number of lanes = 1 | | | | | | | | | | | |
| Ideal condition | 446 | 446 | 446 | 446 | 446 | 446 | 446 | 446 | 446 | 446 | 446 | 446 |
| Access flow (1) | 240 | 265 | 291 | 316 | 341 | 366 | 391 | 416 | 446 | 446 | 446 | 446 |
| Access flow (2) | 390 | 392 | 396 | 401 | 408 | 414 | 421 | 428 | 437 | 442 | 446 | 446 |
| Access flow (3) | 402 | 406 | 410 | 413 | 417 | 426 | 437 | 446 | 446 | 446 | 446 | 446 |
| Access flow (4) | 159 | 203 | 247 | 285 | 319 | 346 | 369 | 397 | 423 | 431 | 446 | 446 |
| Access flow (5) | 188 | 249 | 304 | 352 | 394 | 420 | 437 | 446 | 446 | 446 | 446 | 446 |
| Access flow (6) | 383 | 393 | 402 | 412 | 420 | 428 | 437 | 446 | 446 | 446 | 446 | 446 |
|  | Case 3: upstream access point, the number of lanes = 2 | | | | | | | | | | | |
| Ideal condition | 891 | 891 | 891 | 891 | 891 | 891 | 891 | 891 | 891 | 891 | 891 | 891 |
| Access flow (1) | 736 | 751 | 766 | 781 | 796 | 811 | 825 | 840 | 860 | 870 | 885 | 891 |
| Access flow (2) | 804 | 808 | 811 | 818 | 826 | 835 | 845 | 855 | 869 | 876 | 887 | 891 |
| Access flow (3) | 810 | 811 | 811 | 812 | 813 | 813 | 821 | 836 | 857 | 869 | 885 | 891 |
| Access flow (4) | 428 | 548 | 631 | 691 | 740 | 777 | 802 | 819 | 838 | 852 | 870 | 887 |
| Access flow (5) | 782 | 793 | 803 | 814 | 824 | 834 | 845 | 855 | 869 | 876 | 887 | 891 |
| Access flow (6) | 820 | 827 | 833 | 840 | 843 | 844 | 849 | 858 | 870 | 877 | 887 | 891 |
|  | Case 4: downstream access point, the number of lanes = 2 | | | | | | | | | | | |
| Ideal condition | 891 | 891 | 891 | 891 | 891 | 891 | 891 | 891 | 891 | 891 | 891 | 891 |
| Access flow (1) | 686 | 718 | 749 | 781 | 813 | 844 | 876 | 891 | 891 | 891 | 891 | 891 |
| Access flow (2) | 826 | 826 | 831 | 840 | 850 | 861 | 872 | 884 | 891 | 891 | 891 | 891 |
| Access flow (3) | 855 | 861 | 868 | 857 | 861 | 876 | 891 | 891 | 891 | 891 | 891 | 891 |
| Access flow (4) | 344 | 453 | 550 | 630 | 701 | 758 | 806 | 838 | 864 | 873 | 891 | 891 |
| Access flow (5) | 388 | 512 | 624 | 722 | 806 | 855 | 887 | 891 | 891 | 891 | 891 | 891 |
| Access flow (6) | 833 | 845 | 857 | 869 | 878 | 887 | 891 | 891 | 891 | 891 | 891 | 891 |
|  | Case 5: upstream access point, the number of lanes = 3 | | | | | | | | | | | |
| Ideal condition | 1337 | 1337 | 1337 | 1337 | 1337 | 1337 | 1337 | 1337 | 1337 | 1337 | 1337 | 1337 |
| Access flow (1) | 1182 | 1196 | 1211 | 1226 | 1241 | 1256 | 1271 | 1286 | 1305 | 1316 | 1331 | 1337 |
| Access flow (2) | 1260 | 1263 | 1267 | 1271 | 1277 | 1284 | 1293 | 1303 | 1315 | 1323 | 1333 | 1337 |
| Access flow (3) | 1219 | 1225 | 1232 | 1238 | 1244 | 1250 | 1256 | 1273 | 1297 | 1310 | 1329 | 1337 |
| Access flow (4) | 670 | 859 | 990 | 1086 | 1164 | 1218 | 1253 | 1270 | 1294 | 1304 | 1318 | 1330 |
| Access flow (5) | 1228 | 1238 | 1249 | 1259 | 1270 | 1280 | 1290 | 1301 | 1314 | 1322 | 1332 | 1337 |
| Access flow (6) | 1297 | 1300 | 1304 | 1307 | 1310 | 1314 | 1317 | 1321 | 1327 | 1330 | 1335 | 1337 |
|  | Case 6: downstream access point, the number of lanes = 3 | | | | | | | | | | | |
| Ideal condition | 1337 | 1337 | 1337 | 1337 | 1337 | 1337 | 1337 | 1337 | 1337 | 1337 | 1337 | 1337 |
| Access flow (1) | 1131 | 1168 | 1205 | 1241 | 1278 | 1315 | 1337 | 1337 | 1337 | 1337 | 1337 | 1337 |
| Access flow (2) | 1281 | 1284 | 1287 | 1295 | 1305 | 1316 | 1328 | 1337 | 1337 | 1337 | 1337 | 1337 |
| Access flow (3) | 1300 | 1305 | 1311 | 1316 | 1321 | 1337 | 1337 | 1337 | 1337 | 1337 | 1337 | 1337 |
| Access flow (4) | 499 | 696 | 857 | 994 | 1107 | 1194 | 1256 | 1290 | 1316 | 1323 | 1337 | 1337 |
| Access flow (5) | 592 | 781 | 950 | 1099 | 1225 | 1297 | 1337 | 1337 | 1337 | 1337 | 1337 | 1337 |
| Access flow (6) | 1337 | 1337 | 1337 | 1337 | 1337 | 1337 | 1337 | 1337 | 1337 | 1337 | 1337 | 1337 |
